# Review of cold-induced urticaria characteristics, diagnosis and management in a Western Canadian allergy practice

**DOI:** 10.1186/s13223-018-0310-5

**Published:** 2018-12-18

**Authors:** Peter Stepaniuk, Kateryna Vostretsova, Amin Kanani

**Affiliations:** 1 Department of Internal Medicine, University of British Columbia, Vancouver, BC Canada; 2 Division of Allergy and Immunology, University of British Columbia, Suite 207 - 3195 Granville Street, Vancouver, BC V5R 3V8 Canada

**Keywords:** Cold-induced urticaria, Physical urticaria, Review, Diagnosis, Treatment

## Abstract

**Background:**

Cold-induced urticaria is a significant condition, especially among young females. Despite the morbidity of this disease, studies that fully characterize the disease are limited.

**Methods:**

We analyzed the characteristics of patients diagnosed with cold-induced urticaria at a community-based allergy practice in Vancouver, BC, Canada between 2003 and 2016. Detailed patient history, diagnostic measures and treatment were evaluated.

**Results:**

A total of 50 patients were found to have active cold-induced urticaria with a median age of 28.5 (range 2–67) years and 35 patients (70%) were female. 16 patients (32%) had co-occurring physical urticarias while 26 patients (52%) had secondary allergic diagnoses and 3 patients (6%) were thought to have a provoking factor. Of those with a clinical history of suspected cold-induced urticaria that were evaluated with ice cube testing, a positive test was obtained in 84.7% of patients. Treatment was largely with non-sedating antihistamines, with the majority of patients receiving this modality.

**Conclusions:**

Cold-induced urticaria is a complex disease with significant overlap with other chronic inducible urticarias and other allergic diseases. Diagnostic testing shows inconsistent results and the mainstay of treatment consists of non-sedating antihistamines, with other options available for those who do not respond.

## Background

Cold-induced urticaria is the second most common type of chronic inducible urticaria and has been reported as early as 1866 [1]. It results in pruritic wheals with or without angioedema secondary to the release of leukotrienes, histamine and pro-inflammatory mast cell mediators after exposure of the skin to cold air, liquid or cold objects [2]. Symptoms are typically local and occur minutes after exposure, however severe systemic reactions and anaphylaxis is a risk especially if a large surface area is exposed to the cold such as when swimming in cold water. One retrospective study from Spain and Portugal showed that nearly 20% of their patients with cold-induced urticaria presented with life threatening reactions to the cold [3]. It is a relatively rare disorder with a reported prevalence of 0.05% as per one study in Europe, with a higher prevalence in northern climates [4]. It is also self-limited, lasting 4–5 years on average. However, some studies have suggested that only one quarter of patients will develop resolution of their symptoms within 10 years [5]. The majority of the people affected are young adults and females seem to be affected more frequently [6, 7].

Confusion in the classification and nomenclature of the different forms of urticaria is an ongoing issue. In the 2013 international guidelines, the urticarial diseases were divided into acute (duration of symptoms less than 6 weeks) and chronic (duration of symptoms of greater than or equal to 6 weeks) [8]. Chronic urticaria could then be subdivided into chronic spontaneous urticaria (no identifiable external trigger) and chronic inducible urticaria. The chronic inducible urticarias include the physical urticarias such as cold-induced urticaria [8]. Therefore, by definition patients diagnosed with cold-induced urticaria have a chronic urticaria and must have a duration of symptoms of at least 6 weeks.

Cold-induced urticaria is divided into primary and secondary forms and there have been many associations reported in the literature. Primary forms are defined when a secondary, or triggering, etiology cannot be determined [2]. Secondary cold-induced urticaria has been reported to be associated with both bacterial and viral infections, medications, Hymenoptera stings, hematological malignancies and immunotherapy, largely based on various case reports [9–14]. Diagnosis is made through provocation tests including either the ice cube test or TempTest^®^ measurements, both of which were listed as acceptable in recent guidelines outlined by the 2016 EAACI/GA^2^LEN/EDFUNEV [15, 16]. Treatment is largely symptomatic and consists of cold avoidance and medical management with antihistamines. For those resistant to antihistamines, omalizumab is recommended and has worked well in some trials [17].

Despite its relative predominance throughout those with inducible urticarias, very few studies have fully characterized the disease by analyzing associated physical urticarias, prevalence of possible triggers, association with secondary allergic diagnoses and family history. The goal of this study was to review the current literature available on the subject and to analyze the characteristics of the disease in a cohort of patients in a local allergy clinic to look for possible trends and associations to fuel future research into this field.

## Methods

### Study population

The study was taken in a mixed pediatric/adult practice allergy clinic in Vancouver, British Columbia, Canada operated by two attending physicians. Referrals are generally received by family physicians in addition to some from medical or surgical specialists. A chart review was undertaken to review patient visits from 2003 to 2016 using the electronic medical record system. Files were retrieved by searching the electronic database for the word “cold” and “urticaria”. The authors then manually reviewed the patient charts and included patients with a diagnosis of cold-induced urticaria in the study (see below). If patients had more than one consultation letter, the patient was only included once in the study. Relevant clinical and laboratory data was retrieved from the charts. This included demographic data, other types of co-existing physical urticarias, preceding/causative factors, associated symptoms, secondary allergic diagnoses, family history, laboratory values and treatment regimens.

### Diagnostic criteria

Patients were included in the main analysis if they met the following diagnostic criteria. They must have active symptoms at the time of consultation, duration of symptoms of longer than 6 weeks, test positive on cold provocation testing with the ice cube test and have a clinical history consistent with primarily cold-induced urticaria. Questions on history included the development of urticaria or anaphylaxis on exposure to cold environments, after consumption of cold foods and/or beverages or after immersion in cold water. Only patients considered to have primarily cold-induced urticaria (i.e. their urticarial symptoms were predominately or significantly induced by the cold as opposed to another physical urticaria) where included in the study. Cold provocation testing consisted of applying a melting ice cube in a thin plastic bag to the patient’s volar forearm for 5 min, and then observing for the development of a wheal. A positive test was defined as the formation of a wheal within 10 min after the ice cube stimulus was removed.

The investigation into and diagnosis of other physical urticarias was initiated based on the patient’s clinical history. Dermatographic urticaria was diagnosed using Fric test^®^ instrument. The instrument was placed vertically on the patient’s forearm and stroked once for a distance of approximately 60 mm. The test was considered positive if a pruritic palpable wheal of greater than or equal to 3 mm width was present within 10 min of provocation. Cholinergic urticaria was diagnosed based on the patient’s clinical history. A patient was considered to have a history of cholinergic urticaria if they developed urticarial lesions shortly after exercise, application of heat (such as a hot shower), and/or after consumption of hot and spicy foods or beverages. Provocation testing for cholinergic urticaria did not take place in this allergy clinic.

### Data analysis

Data was analyzed using Microsoft Excel. Patients who met the diagnostic criteria for cold-induced urticaria as outlined above were included in the main analysis. To evaluate the effectiveness of cold provocation testing, we had a sub group of patients we analyzed with a clinical suspicion of cold-induced urticaria. These patients must have undergone provocation testing with a definitive result to be considered for analysis. Patients were considered to have a clinical suspicion of cold-induced urticaria if they had active symptoms at the time of consultation and had a clinical history consistent with primarily cold-induced urticaria (as outlined above).

## Results

### Patient characteristics

50 patients met the diagnostic criteria for cold-induced urticaria. The average age of patients seen at time of consultation was 31.3 years (median age of 28.5) years with a minimum age of 2 years and maximum of 62 years. 30% of the patients were male with 70% being female. Sixteen (32%) patients with cold-induced urticaria had evidence of co-occurring physical urticarias, including cholinergic (10% of patients) and dermographism (22% of patients). Two (4%) of the patients reported associated anaphylaxis from the cold and six (12%) patients had associated angioedema. Just over one half (52%) of patients had secondary allergic diagnoses with allergic rhinoconjunctivitis (34%) and asthma (14%) being the most common (Table 1).

**Table 1:** Patient characteristics in subjects with cold-induced urticaria

	Total patients with cod-induced urticaria (n = 50)
Female: n (%)	35 (70)
Age (years): median (range)	28.5 (2–62)
Co-occurring physical urticarias: n (%)	
Total with co-occurring physical urticaria	16 (32)
Cholinergic	5 (10)
Dermatographic	11 (22)
Cold induced anaphylaxis: n (%)	2 (4)
Cold induced angioedema: n (%)	6 (12)
Secondary allergic diagnoses: n (%)	
Total with other atopic diseases	26 (52)
Allergic rhinoconjunctivitis	17 (34)
Asthma	7 (14)
Atopic dermatitis	3 (6)
Contact dermatitis	1 (2)
Food allergy	2 (4)
Idiopathic anaphylaxis	1 (2)
Insect sting reaction	1 (2)
Medication allergy	1 (2)
Oral allergy syndrome	2 (4)
Suspected preceding/inducting factors: n (%)	
Total with suspected preceding factor	3 (6)
Insect sting	1 (2)
Infection	2 (4)
Family history: n (%)	3 (6)

In total, three patients (6%) were believed to have a preceding factor that may have triggered their cold-induced urticaria. One patient suffered a Hymenoptera sting with acute urticaria, after which the symptoms of cold-induced urticaria began. Two patients (4%) were believed to have their cold-induced urticaria triggered by an infection. The first patient was a 40-year-old male who had two episodes of infectious mononucleosis as a young adult, after which he developed cold-induced urticaria. The other patient was a 24 year-old female who suffered two episodes of pneumonia, and then developed cold-induced urticaria. Neither of these patients had viral loads or serology available to prove the theory. Three patients (6%) reported a family history of cold-induced urticaria (Table 1).

### Cold provocation testing in patients with suspected cold-induced urticaria

A total of 59 patients were identified as having a clinical suspicion of cold-induced urticaria and had cold provocation testing performed using the ice cube test. Of these patients, 84.7% tested positive and 15.3% tested negative.

### Laboratory testing in patients with confirmed cold-induced urticaria

Twenty-two (44%) patients with confirmed cold-induced urticaria had laboratory testing to evaluate for cryoglobulins and/or cold agglutinins. Laboratory blood work was ordered for five patients, but the results could not be located due to either the patient not having blood drawn or the results not received in the allergy/immunology clinic. Of the 17 patients with resulted laboratory values, 16 patients were tested for cryoglobulins and 15 patients were tested for cold agglutinins. All patients tested for cryoglobulins and cold agglutinins had negative results.

### Pharmacologic treatment in patients with cold-induced urticaria

Pharmacologic treatment was prescribed for 49 patients (98%) with active urticaria. Of those patients with prescribed therapy, 22 patients (44%) were on regularly prescribed treatment, whereas 27 (54%) patients were on intermittent/as needed therapy. One patient did not have any prescribed pharmacotherapy and was managed with conservative measures alone. The majority of patients with cold-induced urticaria (90%) used non-sedating antihistamines as part of their treatment plan. Fewer patients were on other pharmacotherapies including omalizumab (2%), H2 receptor antagonists (6%) and sodium cromoglycate (2%). It appeared that patients on these second line therapies did not have control of their symptoms with non-sedating antihistamines alone. About one-third of patients (32%) with prescribed therapy had an epinephrine auto-injector (Table 2).

**Table 2:** Prescribed pharmacologic therapy for patients with cold-induced urticaria

	Total patients with cold-induced urticaria (n = 50)
Patients with prescribed therapy: n (%)	49 (98)
Daily/regular	22 (44)
Intermittent/PRN	27 (54)
Non-sedating antihistamines	45 (90)
Omalizumab	1 (2)
H2 receptor antagonists	3 (6)
Sodium cromoglycate	1 (2)
Epinephrine auto-injector	16 (32)

## Discussion

Consistent with previous studies, we found that cold-induced urticaria is a disease that is more common in younger individuals and more likely to affect females as opposed to males [5–7]. About one-third of patients with cold-induced urticaria also had additional co-occurring physical urticarias with cholinergic urticaria and dermatographic urticaria being evident in this study. These results are comparable to a Finnish chart review that examined 220 patients with cold urticaria in 1985 and identified that 30% of patients with cold-induced urticaria had another co-occurring type of urticaria. They found that 19% of patients had cold-induced urticaria associated with dermatographism, and 7% of patients having cold-induced urticaria also had cholinergic urticaria [18]. This suggests that patients with cold-induced urticaria are more likely to have other types of physical urticarias. There is debate in the prevalence of physical urticarias in the general population with one study estimating underlying prevalence of dermatographism at 2–5% [19]. The prevalence of cholinergic urticaria in the general population is also debated, but one study has suggested that 11% of young adults can have mild disease [20]. Conversely, a recent Korean epidemiological study has estimated that the prevalence of dermatographism and cholinergic urticaria in the general population is 0.12% and 0.025% respectively [21]. As cholinergic urticaria is produced by elevation/reduction of core body temperature, it is also reasonable to see an association between cholinergic and cold-induced urticaria.

We observed in this study that there was a large association between cold-induced urticaria and secondary allergic diseases. About half of the patients with cold-induced urticaria also had another diagnosis of an allergic disease. The most common being allergic rhinoconjunctivitis and asthma. This is in contrast to the abovementioned Finnish chart review of cold-induced urticaria patients where only one quarter of patients had another atopic disease with 15% of patients with atopic dermatitis, 14% with allergic rhinoconjunctivitis and 3% with asthma. One possible reason to explain these discordant findings is the independent rising incidence of atopic disease throughout the world over the past 30 years since this Finnish study was published [22].

In this study, three (6%) of the patients with cold-induced urticaria were felt to have a provoking factor (including infections or insect stings) for their cold-induced urticaria based on clinical history. This would be suggestive of secondary acquired cold-induced urticaria. However, these suspicions were not confirmed on laboratory testing. The remainder of patients were believed to have primary acquired cold-induced urticaria. Historically the majority (96%) of patients with acquired cold-induced urticaria have been shown to have primary cold-induced urticaria, with only rare occurrences of secondary cold-induced urticaria [18]. The most common cause of secondary acquired cold-induced urticaria is primary and secondary cryoglobulinemia (e.g. chronic lymphocytic leukemia, lymphosarcoma, leukocytoclastic vasculitis, hepatitis C virus infection, and angioimmunoblastic lymphadenopathy) based on case reports [23–28]. Infectious diseases are the second most common type of secondary acquired cold-induced urticaria [29]. Many case reports have suggested a relationship between secondary cold-induced urticaria and syphilis, rubeola, hepatitis, respiratory viral infections, HIV, and infectious mononucleosis [11, 12, 30, 31]. In our study, we experienced two patients with an infection (Epstein–Barr virus and pneumonia) that were believed to be the triggering factors for those patient’s cold-induced urticaria based on the temporal relationship of their symptoms. There has also been a number of case reports of cold-induced urticaria after insect stings, predominately Hymenoptera stings [9, 32]. We identified one patient in our study who was believed to have developed cold urticaria after an insect sting.

In addition to acquired cold-induced urticaria, there are types of cold-induced urticaria that are felt to have a genetic component with familial transmission. This includes the cryopyrin-associated periodic syndrome (CAPS) which is a rare inherited autoimmune disorder with a mutation in *NLRP3* (*CIAS1*) gene that leads to overproduction of interleukin-1-beta (IL-1β). CAPS consists of three disorders including familial cold autoinflammatory (or urticaria) syndrome (FCAS/FCU), Muckle–Well syndrome (MWS) and neonatal-onset multisystem inflammatory disease (NOMID) also known as chronic infantile neurologic cutaneous articular syndrome (CINCA) [33]. Diagnosis of CAPS can be difficult and relies on genetic testing for *NLRP3* and other inflammatory diseases along with full evaluation of symptoms, lab tests (inflammatory markers, serum amyloid A), skin biopsy and complete medical history [34]. In our study, we identified three patients (6%) with a notable family history of cold-induced urticaria, however no patients were suspected of having CAPS in this study as they did not have other features of this syndrome and responded well to antihistamine therapy. Interestingly, Neittannmaki et al. [18] also reported that 4.5% of patients in that study had a family history cold-induced urticaria suggesting that there may be a genetic component of the primary acquired cold-induced urticarias.

Newly released consensus guidelines for the diagnosis and treatment of cold-induced urticaria were recently published in 2016 [16]. It is recommended that diagnosis should be made with the aid of provocation tests in order to obtain a definitive diagnosis. Several provocation tests are available including ice cube tests, cold water baths or TempTest^®^ measurements. Although there is no study comparing the sensitivity and specificity of each of these tests, both the ice cube test and TempTest^®^ are considered acceptable for testing [16]. Conversely, some studies have suggested that the TempTest^®^ may be a better test as it allows for reproducible and standardized cold and hot provocation tests along with identification of temperature and stimulation time thresholds [16]. However, the specificity of these testing methods has been debated with one study suggesting that about one quarter of patients with cold-induced urticaria will have a negative cold stimulation test [3]. In our study, we found that of the 59 patients that had a high likelihood of cold-induced urticaria based on clinical history, 15.3% had a negative test. This may reflect the poor sensitivity of ice cube testing as a modality and questions the utility of using provocation testing in making the diagnosis.

Given that most cases of cold-induced urticaria are idiopathic, targeted treatment is often not possible. Avoidance of cold, such as immersion into cold bodies of water, is often recommended for both treatment and avoidance of a potentially more systemic reaction such as anaphylaxis. However, this advice is often not achievable or fully effective in managing symptoms [2]. Non-sedating antihistamines, used up to four times the standard dose for those that do not respond to the standard dose, has been shown in multiple clinical trials to be effective in controlling the frequency and severity of symptoms associated with cold-induced urticaria, regardless of the etiology [35–40]. For most patients, antihistamines alone are effective and we found that nearly all patients in our study were on a non-sedating antihistamine as part of their treatment regimen. The choice of daily versus intermittent dosage is based on the severity of symptoms and for those patients with mild symptoms, intermittent use of antihistamines or simply cold avoidance alone may suffice. For patients with refractory symptoms on maximal treatment with antihistamines, treatment with omalizumab or cyclosporine is recommended. A small randomized placebo-controlled trial by Metz et al. [17] showed that omalizumab was effective in reducing overall disease activity at both the 150 mg and 300 mg doses. One patient in our study was on omalizumab. Omalizumab is currently indicated for the management of chronic spontaneous urticaria and its use for the inducible urticaria is considered off label [41]. The use of cyclosporine in cold induced urticaria is based on case reports and its success in the treatment of chronic spontaneous urticaria [42]. A number of case reports and small randomized control trials have also showed success in treatment of cold urticaria with antibiotic therapy (including penicillin and doxycycline), H2 antihistamines such as ranitidine, leukotriene antagonists, etanercept and tricyclic antidepressants such as Doxepin [43–49]. These second line agents are generally used in conjunction with other medications, such as antihistamines, in patients with difficult to control symptoms.

## Conclusions

Cold-induced urticaria is a complex disease with significant overlap with other chronic inducible urticarias. A higher rate of atopic disorders has also been reported in this study which has not been reported by others. Idiopathic primary cold-induced urticaria continues to be most prevalent, however secondary causes such as due to cryoglobulinemia, infectious agents and insect stings can be identified in a cohort of patients. Although rare, in those with a family history of cold induced urticaria, a diagnosis of CAPS and other familial disorders should be entertained. Despite multiple different testing modalities currently available, diagnostic testing has been shown to have inconsistent results. The mainstay of treatment consists of cold avoidance techniques with non-sedating antihistamines being the most common pharmacotherapy employed. Other agents, including omalizumab and cyclosporine, are also available for those who do not respond to initial management. Although, this was a small chart review, we feel that this study adds to the growing body of knowledge that currently exists on cold-induced urticaria and reviews the current literature available to aid in diagnosis and management of this disease.
